# The dilatable membrane of oleosomes (lipid droplets) allows their *in vitro* resizing and triggered release of lipids†

**DOI:** 10.1039/d3sm00449j

**Published:** 2023-08-05

**Authors:** Eleni Ntone, Benjamin Rosenbaum, Simha Sridharan, Stan B. J. Willems, Othonas A. Moultos, Thijs J. H. Vlugt, Marcel B. J. Meinders, Leonard M. C. Sagis, Johannes H. Bitter, Constantinos V. Nikiforidis

**Affiliations:** a Biobased Chemistry and Technology, Wageningen University and Research Bornse Weilanden 9, PO Box 17 6708 WG Wageningen The Netherlands costas.nikiforidis@wur.nl +31(0)317488042; b TiFN P.O. Box 557 6700 AN Wageningen The Netherlands; c Engineering Thermodynamics, Process & Energy Department, Faculty of Mechanical, Maritime and Materials Engineering, Delft University of Technology Leeghwaterstraat 39 2628 CB Delft The Netherlands; d Laboratory of BioNanoTechnology, Wageningen University and Research, Axis Bornse Weilanden 9 6708 WG Wageningen The Netherlands; e Food and Biobased Research, Wageningen University and Research Centre P.O. Box 17, Bornse Weilanden 9 6708 WG Wageningen The Netherland; f Laboratory of Physics and Physical Chemistry of Foods, Wageningen University Bornse Weilanden 9 6708 WG Wageningen The Netherlands

## Abstract

It has been reported that lipid droplets (LDs), called oleosomes, have an inherent ability to inflate or shrink when absorbing or fueling lipids in the cells, showing that their phospholipid/protein membrane is dilatable. This property is not that common for membranes stabilizing oil droplets and when well understood, it could be exploited for the design of responsive and metastable droplets. To investigate the nature of the dilatable properties of the oleosomes, we extracted them from rapeseeds to obtain an oil-in-water emulsion. Initially, we added an excess of rapeseed oil in the dispersion and applied high-pressure homogenization, resulting in a stable oil-in-water emulsion, showing the ability of the molecules on the oleosome membrane to rearrange and reach a new equilibrium when more surface was available. To confirm the rearrangement of the phospholipids on the droplet surface, we used molecular dynamics simulations and showed that the fatty acids of the phospholipids are solubilized in the oil core and are homogeneously spread on the liquid-like membrane, avoiding clustering with neighbouring phospholipids. The weak lateral interactions on the oleosome membrane were also confirmed experimentally, using interfacial rheology. Finally, to investigate whether the weak lateral interactions on the oleosome membrane can be used to have a triggered change of conformation by an external force, we placed the oleosomes on a solid hydrophobic surface and found that they destabilise, allowing the oil to leak out, probably due to a reorganisation of the membrane phospholipids after their interaction with the hydrophobic surface. The weak lateral interactions on the LD membrane and their triggered destabilisation present a unique property that can be used for a targeted release in foods, pharmaceuticals and cosmetics.

## Abstract 2

It has been reported that lipid droplets (LDs), called oleosomes, have an inherent ability to inflate or shrink when absorbing or fueling lipids in the cells, showing that their phospholipid/protein membrane is dilatable.

## Graphical abstract

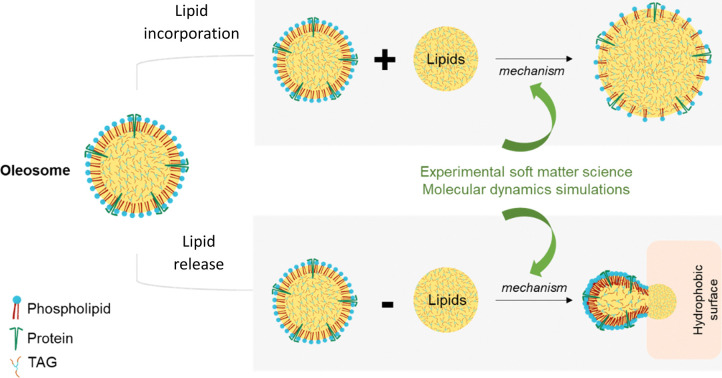

## Introduction

Lipid droplets (LDs), also known as oleosomes, when derived from seeds, are ubiquitous cell organelles increasingly acknowledged in cell biology as dynamic molecular machinery.^1^ During their biogenesis, triacylglycerols are coated with a monolayer of phospholipids (PLs) decorated with proteins.^2–5^ A major contribution of oleosomes is cell homeostasis, which is realized through lipid trafficking; oleosomes absorb free lipids to prevent lipotoxicity and regulate lipid supply,^6^ act as hubs of lipid synthesis and accumulation,^7–11^ or supply lipids to other cell organelles^9–11^ and even invasive microorganisms.^12^

During the highly controlled *in vivo* processes of the uptake or release of lipids, oleosomes can deflate or shrink.^9–11^ Enzymatic reactions play a key role in the lipid uptake and release mechanism; however, the ability of oleosomes to deflate or shrink could be attributed to the mechanical properties of their membrane.^9,10,13,14^

Inspired by the fascinating properties of oleosomes *in vivo*, we aim to purify them from rapeseeds and investigate purely the mechanical properties of their interface, when the biological processes, like enzymatic reactions, are excluded.

The extent of the lateral interactions in the oleosome membrane is still unknown; therefore, we used a combination of experimental techniques with molecular dynamics simulations to investigate it. With this knowledge, we aim to investigate the mechanical properties of the oleosome membrane and understand whether the phospholipids and proteins are forming clusters or are “solubilized” and mobile on the interface. Additionally, we also aim to understand whether the strength of lateral interactions could be used to destabilise the oleosomes and release the internal oil.

## Materials and methods

### Materials

The oleosomes were extracted from untreated Alize rapeseeds stored at −18 °C. Rapeseed oil was kindly provided by Nutricia Research B.V. and was striped using dry silica before further use to remove polar compounds. All chemicals used were of analytical grade and were purchased from Sigma Aldrich (St Louis, MO, USA).

### Purification of rapeseed oleosomes

Pure oleosomes were extracted using the protocol as described by de Chirico *et al.* (2018)^15^ with the following modifications: 100 g of dehulled rapeseeds were soaked in a solution of sodium bicarbonate with a pH of 9.5 (0.1 M) and the seeds to buffer ratio of 1 : 7 w/w for 4 hrs under continuous stirring (RW 20 digital stirrer, IKA®, Staufen, Germany) to ensure proper mixing. After soaking, the seeds were blended at maximum speed for 90 seconds (Philips Avance HR2093, Eindhoven, the Netherlands). As a first step to removing the solids, the mixture was passed through a cheesecloth. The filtrate was centrifuged (30 min.; 10 000 g; 4 °C; SORVALL Legend XFR centrifuge by Thermo Fischer SCIENTIFIC, Waltham, USA) in 250 mL centrifuge tubes to remove extraneous proteins and fibres. After centrifugation, the top layer (cream) was collected. The cream layer was spread over a filter paper (Whatman®, grade 4) to absorb most of the remaining liquid. The cream was resuspended in a new extraction medium (1 : 4 w/w) and centrifuged under the same conditions. After the second centrifugation, the cream layer was collected in the same manner and resuspended in deionized water (1 : 4 w/w). After the third and final centrifugation, the cream layer containing the purified oleosomes was again collected and then stored at 4 °C until further use.

### Homogenization of purified oleosomes with free lipids

The purified oleosomes were dispersed in deionized water to a final concentration of 10 wt% oleosomes. Seventy (70) g of oleosome dispersion was first sheared using a disperser (Ultra-Turrax, IKA®, Staufen, Germany) at 8000 rpm for 30 s. Next, rapeseed oil was slowly added to the dispersion in a mass ratio of 1 : 1 or 1 : 3 of oleosomes in the dispersion (*i.e.* 7 g) to rapeseed oil (7 and 21, respectively) and sheared for 1 min at 10 000 rpm. The formed coarse emulsion was further processed using a high-pressure homogenizer (GEA®, Niro Soavi NS 1001 L, Parma, Italy) for 5 cycles at 300 bars. The number of experiments and additional analyses was at least n ≥ 3 of independent experiments.

### Particle size distribution of oleosomes before and after free lipid absorption

The particle size distribution of the oleosomes after treatment was determined by laser diffraction using a Bettersizer S3 Plus instrument (3P Instruments GmbH & Co. KG, Odelzhausen, Germany). The measurement settings were adjusted to a refractive index 1.46–1.47 and a density 0.91 g cc^−1^ for rapeseed oleosomes. 1.0 wt% Sodium dodecyl sulfate (SDS) was added to the samples in a ratio of 1 : 1 (v/v). SDS is a low molecular weight surfactant whose role is to break protein hydrophobic interactions^16^ and it was added to our system to prevent bridging between the oleosomes. This allowed us to determine the actual individual droplet size instead of the aggregates. The stirring speed of the small-volume sample dispersion unit was set to 1600 rpm. The measurements were reported as volume mean diameter (*d*_4,3_ = Σ*n*_i_*d*_i_^4^/Σ*n*_i_*d*_i_^3^) and surface mean diameter (*d*_3,2_ = Σ*n*_i_*d*_i_^3^/Σ*n*_i_*d*_i_^2^), where *n*_i_ is the number of droplets with a diameter of *d*_i_. The average values are a result of measurements of at least three individual samples (*n* ≥ 3) and the ± symbol represents the standard deviation.

### Calculation of surface area and estimation of molecules per surface area before and after free lipid absorption by oleosomes

The total area (*A*_T_) of the oleosomes before and after contact with the clustered hydrophobic molecules (TAGs) was calculated using the equation:

1 *A*_T_ = *A*_d_·*N*_d_

where *A*_d_ is the area of one droplet and is equal to 
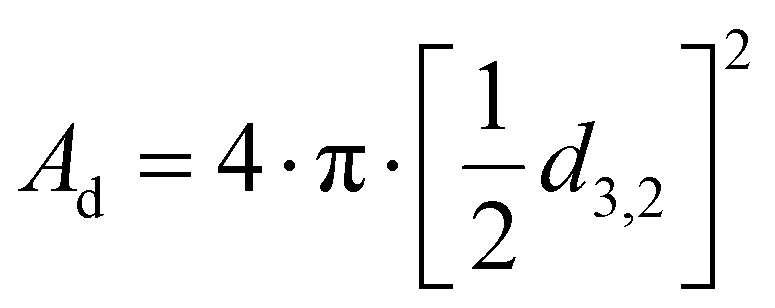
*, N*_d_ is the number of droplets, equal to 
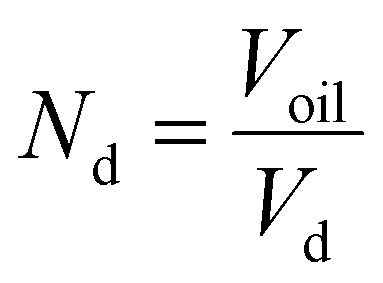
, *V*_d_ is the volume of one droplet, equal to 
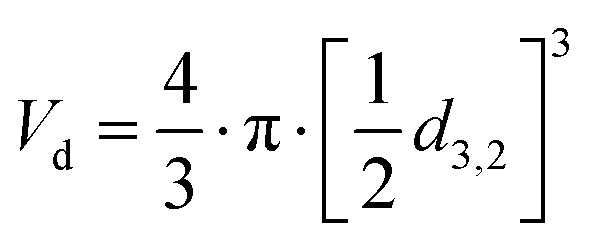
.

The number of phospholipids (*N*_PL_) per area was estimated using the equation:2
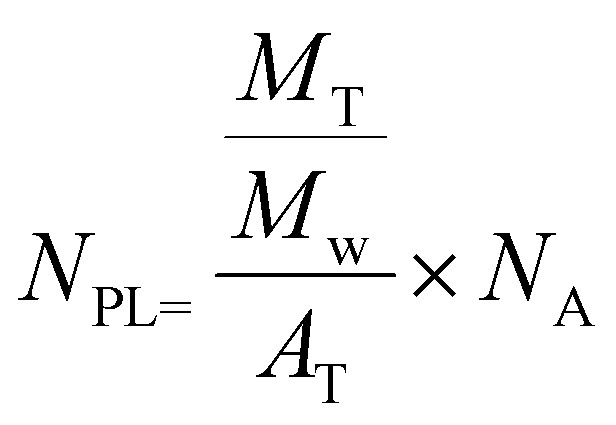


*M*_T_ represents the total mass of phospholipids in the reference oleosome dispersion, assuming that it constitutes 0.6 wt% of the total oleosome mass.^17^ Phospholipids (PLs) in seed oleosomes account for around 0.6–2 wt%^17–19^ while proteins account for 0.4–1.4 wt% of the total oleosome mass.^18,20^ We used the PL as an example, but the same trend is expected for proteins associated with oleosomes.

*M*_w_ is the molecular weight of the PL. Assuming that these are all phosphatidylcholine, *M*_w_ is equal to 786.1 g mol^−1^ and *N*_A_ is Avogadro's number (6.02214 × 10^23^ mol^−1^). The inverse quantity [1/*N*_PL_] gives the area per PL.

### Confocal laser scanning microscopy

The structure of oleosomes before and after free lipid absorption was studied using a confocal laser scanning microscope (Leica SP8-SMD microscope, Leica Microsystems, Wetzlar, Germany) with a 63× magnification water immersion lens. The LD samples after lipid absorption were diluted 100× in deionized water. Nile Red (0.01 wt% in ethanol) was used to stain the lipids in a ratio of 1 : 200 (v/v) dye solution to the sample. The samples were excited at *λ* = 488 nm using a white light laser source. The images were analyzed using the Leica Application Suite X software.

### Dilatational interfacial rheology

To apply dilatational interfacial rheology, the oleosome interface was reconstructed in a drop tensiometer using the isolated molecules present in the oleosome interface of the purified rapeseed oleosomes. First, the purified oleosomes were dried in an oven at 45 °C for 2 days. The dried oleosomes were defatted using Soxhlet extraction with petroleum ether for 7 h. The remaining solids (molecules from the oleosome interface) were left under the fume hood for solvent evaporation for 2 days. The solids were ground to a fine powder using a mortar and stored at −18 °C until further use. Dispersions containing 0.05–0.001 wt% of the isolated oleosome interfacial molecules were prepared in deionized water in a conical glass flask. To ensure solubilization, each dispersion was subjected to an ultrasonication bath for 1 h before the measurements. Oscillatory dilatational interfacial rheology was applied to characterize the interfacial elastic (
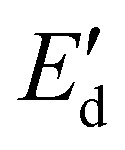
) and viscous (
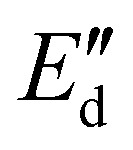
) moduli as a function of deformation amplitude using an automated drop tensiometer (ADT, Tracker, Teclis instruments, Tassin, France). The dispersions were transferred to the cuvette, the syringe was immersed and 15 min of waiting time was applied for any insoluble material to settle down and any air bubbles created from the sonication to reach the surface. Thereafter, the first oil droplet was expelled and a new oil droplet with a surface area of 20.0 mm^2^ was created at the tip of a rising-drop capillary needle (gauge 20). Stripped rapeseed oil was used. The interfacial tension *γ* was calculated from the shape of the droplet using the Laplace equation and monitored for 2 h (7200 s) at 20 °C. Later, the droplet was subjected to sinusoidal deformations with an amplitude of 5–50% of its original surface area at a constant frequency (0.02 Hz). Each amplitude consisted of a series of 5 cycles followed by a period of 5 blank cycles. The interfacial tension and area changes were recorded during oscillations, and the dilatational elastic (
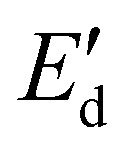
) and viscous (
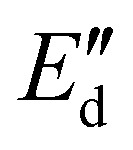
) moduli were obtained according to the equations:3
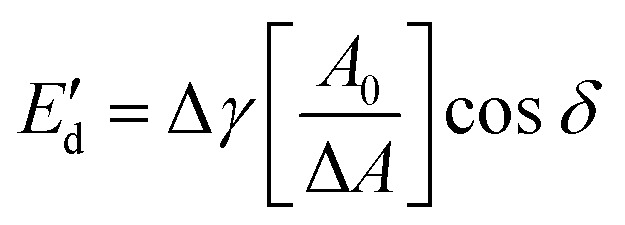
4
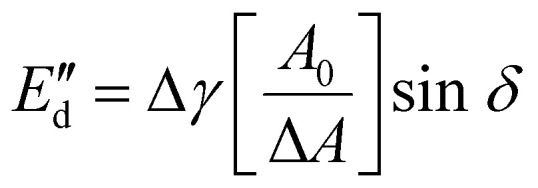
where Δ*γ* is the change of interfacial tension at each deformation, *A*_0_ is the initial droplet surface area (20.0 mm^2^), Δ*A* is the change in the droplet surface area and *δ* is the phase shift oscillatory interfacial tension signal. The results are an average of two individual samples (*n* = 2).

### Contact of purified oleosomes with hydrophobic surfaces

**Fig. 1:**
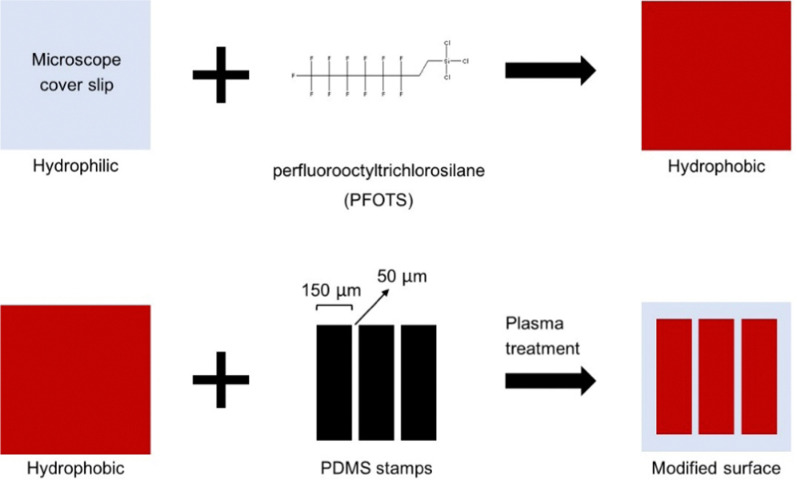
Flow diagram for the preparation of surfaces with alternating hydrophobic and hydrophilic lanes.

Fluorescent microscope imaging was employed to study the contact establishment of oleosomes with surfaces of different hydrophobicities. A patterned surface consisting of hydrophobic and hydrophilic lanes was prepared according to previously published work.^21^ The surface was modified with 1*H*,1*H*,2*H*,2*H*-perfluorooctyl trichlorosilane (PFOTS), which is hydrophobic. First, microscope coverslips were cleaned in ethanol and water and then dried. Coverslips were then placed in a plasma oven for 5 minutes at high energy to ‘activate’ the surface and generate free silanol groups (this allows for the trichlorosilane part to form an assembled monolayer on the glass surface). The coverslips were then placed in a glass Petri dish in a desiccator along with a glass vial containing 100 μL of pure PFOTS (PFOTS is therefore not directly in contact with coverslips). The desiccator was then pumped down to a high vacuum, the pump was switched off and the desiccator was left overnight for chemical vapor deposition (CVD) of PFOTS on the coverslips (a general method for creating coatings on (glass) surfaces). The next day, the slides were removed from the desiccator, washed with isopropanol, dried, and left in the oven at 70 °C for at least 30 min. For creating patterns, PDMS stamps with line features were cut to an appropriate size (around 0.75 cm^2^), sonicated in ethanol, and then placed on the PFOTS glass surface. Plasma treatment for 4 cycles of 1 min each was then applied to remove any unprotected areas of the surface. A schematic representation of the preparation process of the surfaces is given in Fig. 1. As a reference complete hydrophilic surface, plain microscope coverslips were used which were plasma treated accordingly to remove any impurities.

The dispersions of 10.0 wt% oleosomes were diluted 100 times in deionized water and Nile red was added to stain the lipid phase. The functionalized surfaces were fully covered with the oleosome dispersions for 10 min. Thereafter, the surfaces were gently rinsed with deionized water to remove the excess oleosomes and dried under nitrogen at very mild flow rates. The surfaces were then visualized under a fluorescent light microscope (Leica DMi8, Leica Microsystems, Wetzlar, Germany). To exclude any effect of the added fluorescent dye on LD contact establishment with the surfaces, samples without the addition of dye were also prepared and studied under the light microscope.

Pure hydrophobic (non-patterned) and hydrophilic surfaces were used to determine their contact angles (drop tensiometer). A drop of 5 μl deionized water was placed on the corresponding surface and the contact angle was measured using the single measurement option for a rising drop in a drop tensiometer (ADT, Tracker, Teclis-instruments, Tassin, France). The values were found to be 102° and 60° for the hydrophobic and the hydrophilic surface, respectively.

### Atomic force microscopy

For simplicity in the analysis, non-patterned modified hydrophobic surfaces were used to study the outcome of oleosome contact with hydrophobic surfaces using atomic force microscopy. Oleosome dispersions were prepared as mentioned before without the addition of Nile red and the same procedure of incubation and rinsing was followed. The surfaces were imaged using an atomic force microscope (AFM, MultiMode 8-HR, Bruker, USA). A scanasyst (R) air probe was used with a tip diameter of 2 nm. The Bruker scanasyst tapping mode was used and an image with a scan size of 12.5 × 12.5 μm, a scan rate of 0.977 Hz and 256 lines was used. The images were analysed using Nanoscope Analysis 1.5 software (Bruker, USA).

### Molecular dynamics simulations

**Fig. 2:**
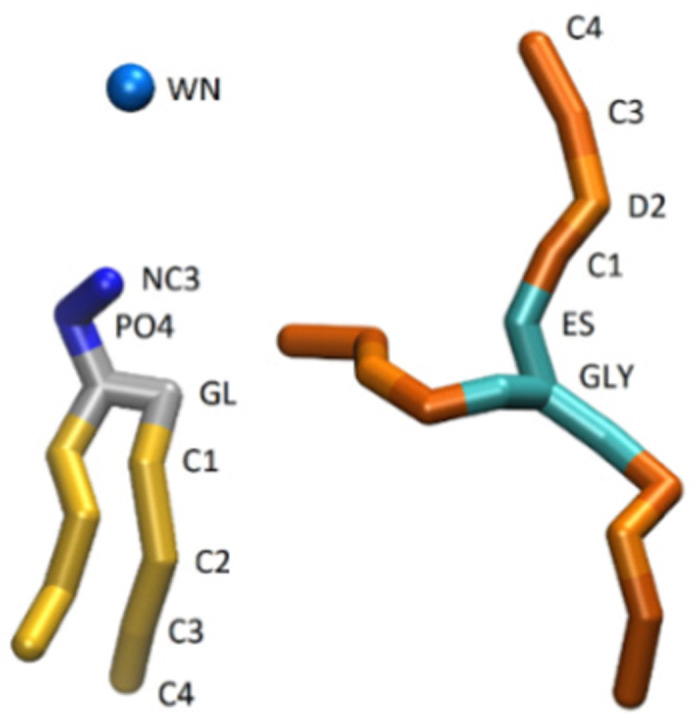
The molecules simulated in this study using the coarse-grained Martini force field.^22^ Left: Water and DPPC, right: triolein. In this study, the number of water, DPPC and triolein molecules used was equal to 350 000, 1200, and 2560, respectively. Coloring scheme: PL glycerol groups (grey), PL head groups (blue), PL lipid groups (yellow), TAG glycerol groups (cyan), and TAG hydrophobic tails (orange).

The open-source MD simulation package GROMACS 2020.2^22^ was used for all simulations. Newton's equations of motion were integrated using the leap-frog algorithm with a timestep of 20 fs. The coarse-grained Martini 3 force field^23^ was used to model all components. Most simulations were conducted on 28 cores achieving simulation speeds of *ca.* 300 ns per day. The cutoff for the van der Waals and electrostatic interactions was set to 1.1 nm. VMD 1.9.3^24^ was used for all visualizations. Our initial system consisted of an oleosome-like droplet made of a triolein core and DPPC as the single PL on the oleosome interface and a similar size droplet of assembled triolein molecules. Both the oleosome-like and the assembled triolein droplet contained 2560 triolein molecules and had radii of approximately 11 nm (Table S1, ESI†). The PL density at the oleosome interface was varied by using 1200, 1600, and 2000 DPPC molecules, corresponding to the PL densities of 0.7, 0.9, and 1.1 PL nm^−2^, respectively. These densities correspond to the mass range of PL in seed oleosomes (0.6–2 wt%).^17–19^ Radii and PL densities of all droplets can be found in Table S1 (ESI†). All initial configurations were created using an in-house python code which allowed us to vary the PL density at the oleosome interface at will. We verified that equivalent initial configurations can be created with open-source software such as PACKMOL.^25^ The simulation box dimensions were set to 52 × 32 × 32 nm. Periodic boundary conditions were imposed in all directions. The molecules from the Martini representation comprising the system were water (WN) as the solvent, DPPC as the single PL covering oleosomes, and triolein as the lipid inside the oleosomes and in the free triolein droplet. The triolein molecule for Martini was constructed based on previously published data,^26^ same for the force field details for the rest of the molecules.^23^Fig. 2 shows the Martini representation of the simulated molecules and the group (bead) names corresponding to the GROMACS input files provided in the ESI.†

The initial configurations comprised two subsystems, each one containing a simulation box with a hydrated TAG and oleosome droplet, respectively. These two boxes were positioned next to each other along the *x*-axis, separated by 0.47 nm. 400 ps–2 ns runs at 295 K and 1 bar in the isothermal isobaric ensemble (*NpT*) were carried out to homogenize the two subsystems and equilibrate the final system. Production runs of 50 ns–1 μs at 305 K and 1 bar in the *NpT* ensemble were performed for simulating the fusion of the droplets. The Berendsen^27^ and Parrinello–Rahman^28^ barostats with coupling constants of 12 ps were used for the equilibration and production runs, respectively. The velocity rescale thermostat^29^ implemented in GROMACS with a coupling constant of 1 ps was used in all runs. All the GROMACS input files, necessary for reproducing our simulations, are provided in the ESI.† These files were created following the instructions of the Martini Force Field website.^30^ To obtain statistics for the fusion phenomena, ten independent production runs were performed, each one starting from a different initial configuration. It is important to note that the melting temperature of DPPC described by the Martini force field is lower than 283 K,^31^ which is substantially lower than the experimentally measured melting temperature (314 K). Thus, in all simulations performed here, DPPC remained in the liquid phase, and no aggregation or clustering of the DPPC molecules on the surface was observed.

For the computation of radial component densities (RCDs), 200 concentric bins were used. The RCDs are averaged over the number of beads and timeframes, where timeframes were sampled every 10 ps, and divided by the number of frames and the volume of the bin to obtain the component density with a unit of nm^−3^. At least 10 ns of simulation time were used for the computation of RCDs.

The standard deviation of a binomial sampling distribution *p̂* can be calculated from5
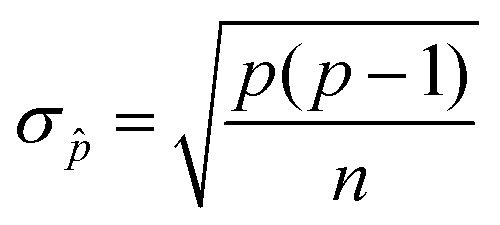
where *p* is the population proportion and *n* is the sample size.

## Results and discussion

In this work, we investigated the extent of the lateral interactions between the molecules in the oleosome membrane. For that, we combined experimental soft matter science, including light scattering, advanced microscopy, surface functionalization, and interfacial dilatational rheology, with coarse-grained molecular dynamics (MD) simulations.

### The molecules of the oleosome membrane can rearrange to cover more surface

**Fig. 3:**
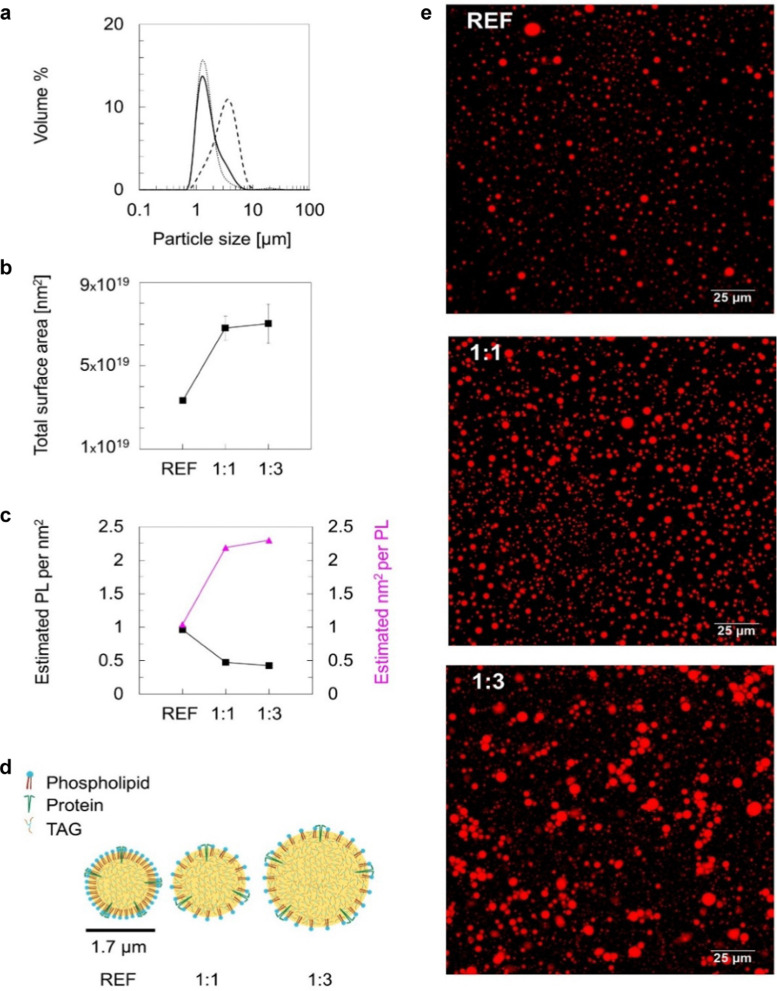
(a) Individual droplet size distribution of oleosomes as a volume% at reference state (continuous line) and after oil addition in the oleosome dispersion at a ratio of 1 : 1 (dotted line), 1 : 3 (dashed line), (b) total surface area of oleosomes as a function of increasing oil mass, (c) estimated number of PL per surface area (black square symbol) and estimated area per PL (magenta triangle symbol) as a function of increasing oil mass, (d) schematic representation of the interface after increasing the available surface (molecules not to scale), (e) CLSM images of oleosomes at reference state (REF) and after oil addition in a mass ratio of 1 : 1 and 1 : 3 stained with Nile Red. Scale bar: 25 μm.

To investigate the ability of oleosomes to adjust the density of the molecules at their interface, we first purified oleosomes from rapeseeds (*Brassica napus*). The oleosomes were recovered as a concentrated cream that contained 24.2 ± 2.8 wt% moisture. Lipids accounted for 95.8 ± 0.9 wt% of the total dry weight, while proteins accounted for 2.0 ± 0.1 wt% of the total dry weight. The protein profile of oleosomes showed the presence of mainly oleosins at the interface (Fig. S1, ESI†), which are the main structural proteins related to rapeseed oleosomes.^32,33^ The oleosomes were thereafter dispersed in deionized water at a final concentration of 10.0 wt%. No coalescence was observed during the storage of the emulsions for 7 days, showing that the oleosome membrane provided sufficient protection. The density of the membrane probably plays a key role in stability against flocculation, therefore we decreased it by adding “free” oil (mostly triacylglycerols-TAGs) and homogenized it to allow the membrane molecules to redistribute and reach a new equilibrium. To investigate the probable redistribution of the oleosome membrane after homogenization with excess oil, we first determined the particle size distribution of the droplets before and after the incorporation of the excess oil (Fig. 3(a)). The initial size distribution of oleosomes ranged from 0.5 to 10.0 μm with an average individual particle size (*d*_4,3_) of 1.7 ± 0.1 μm, which is within the size range generally reported for cytoplasmic oleosomes.^5,34^ By adding free TAGs in the dispersion in a 1 : 1 mass ratio with oleosomes, we observed that there was almost no change in the size distribution (*d*_4,3_ = 1.7 ± 0.4 μm), suggesting that the number of droplets was about two times higher. This result shows that the number of molecules initially present in the oleosome membrane was more than sufficient and could redistribute to stabilize the additionally created surface. Further increase in the mass of added free TAGs (1 : 3 oleosomes:TAGs) led to a shift in the size distribution to higher values and a significant increase of the droplet size, with the *d*_4,3_ value increasing from 1.7 ± 0.1 to 3.3 ± 0.5 μm. Despite the initial droplet coalescence observed, the emulsions were stable after storage for 7 days, showing an efficient redistribution of the membrane molecules and a new equilibrium on the interface, which could prevent further coalescence.

To assess the changes in interfacial density upon the incorporation of additional TAGs in the oleosome emulsion, we roughly calculated the number of molecules per area of oleosomes before and after the addition of free oil. For this calculation, we first determined the total droplet surface area before and after lipid absorption using eqn (1) (Methods), as illustrated in Fig. 3(b). Secondly, we presented the changes in interfacial density as changes in the number of PL per area or area per PL using eqn (2) (Methods), as shown in Fig. 3(c). For simplicity, we focused the discussion of this manuscript on PL, however, since proteins (*i.e.* oleosins) are also present on the interface, we expect a similar trend.

The initial aqueous dispersion of 10.0 wt% oleosomes resulted in an initial total surface area of *ca.* 3 × 10^19^ nm^2^ (Fig. 3(b)). Taking into account the literature information that about 0.6 wt% of rapeseed oleosomes are PL,^17^ we calculated that the interface had approximately an initial density of 1 PL per 1 nm^2^ (or a corresponding area of 1 nm^2^ per PL) (Fig. 3(c)), which is rather dense compared to 1 PL per 6 nm^2^ that molecular simulations have been predicting for the lipid droplet monolayer.^35^ Our calculations are based on an average droplet size of a rather wide droplet size distribution, so it is possible that the available surface is larger than the one we used. Nevertheless, the aim of this work was not to estimate accurately the surface density, but to investigate the interactions between the membrane molecules. To investigate the ability of the membrane molecules to rearrange and cover the new available surface, we added oil in the system at a volume ratio 1 : 1 with the oleosomes and applied high pressure homogenization. The particle size distribution and average droplet size hardly changed (*d*_4,3_ = 1.7 ± 0.4 μm), whereas we hypothesise that the surface area was approximately about two times larger (7 × 10^19^ nm^2^). As the number of PL present in the dispersion was constant, the interfacial density of the newly formed oleosomes is now approximately half of the reference system which for the sake of our calculations we hypothesize was 0.5 PL nm^−2^*vs.* 1 PL nm^−2^. This monolayer density corresponds to 2 nm^2^ available per PL if it was 1 nm^2^ per PL in the reference system, suggesting the formation of oleosomes with surface voids, as has been suggested already for the lipid droplets.^35^ The reason that 1 PL nm^−2^ and 0.5 PL nm^−2^ result in the same droplet size shows that in both cases, the amount of PL on the interface is very high and enough to stabilise the oil droplets with a diameter of about 1 μm. As reported, a PL density on the droplet surface of about 0.1–0.2 PL mn^−2^ is already sufficient.^36^ At the lowest oleosome:oil ratio (1 : 3), the resulted particle size (*d*_4,3_ = 3.3 ± 0.5 μm) led to a similar total surface area (7 × 10^19^ nm^2^) with a similar PL density (0.4 PL nm^−2^), corresponding to an available area of approximately 2 nm^2^ per PL (Fig. 3(b) and (c)). Despite the lower density on the droplet surface, the droplets were still stable against coalescence, showing a homogeneous distribution of the membrane molecules and an effective surface coverage, even after storage for 7 days. A schematic representation of the estimated oleosome interfacial density after free oil absorption is given in Fig. 3(d).

Given that oleosomes could incorporate additional oil in their structure that is even 3 times of their initial lipid mass, is a clear indication that the intermolecular interactions in the interface are weak and the interfacial molecules can be disrupted. The individual droplet size of the oleosomes before and after lipid absorption was also confirmed using confocal microscopy (Fig. 3(e)), which showed similar droplet sizes as measured using light scattering techniques.

These experiments ratify the weak lateral interactions in the oleosome membrane and their ability to redistribute on an available interface. To get more insights into this property, we investigated the viscoelastic properties of the interfacial membrane formed.

### The dilatable interface of oleosomes can reversibly expand and shrink

**Fig. 4:**
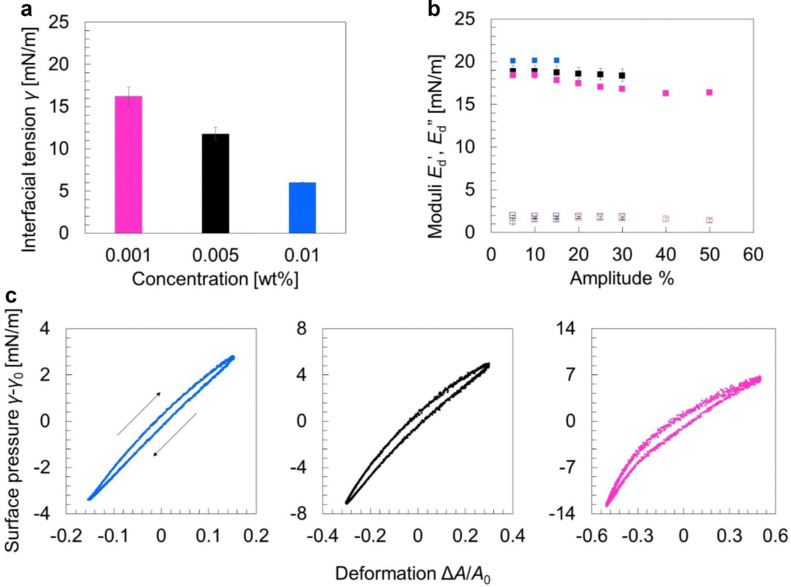
(a) Interfacial tension *γ* of an oil/water interface measured after 2 h as a function of the concentration of oleosome membrane molecules in the aqueous phase (in wt%), (b) dilatational elastic modulus (
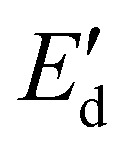
: filled symbol) and viscous modulus (
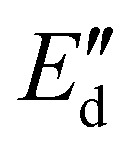
: hollow symbol) as a function of deformation amplitude (10–50%) at constant oscillatory frequency 0.02 Hz, (c) Lissajous plots showing changes in the surface pressure (*γ* − *γ*_0_) upon area deformation (Δ*A*/*A*_0_) at the highest amplitude achieved for each membrane concentration. The curves include the 3 middle cycles of each oscillation. The arrow pointing to the right indicates expansion and the arrow pointing to the left designates compression. The color of the curves corresponds to the colors used for the concentrations in (a).

To investigate the lateral molecular interactions in the oleosome interface and its viscoelastic properties during expansion and shrinkage, we used interfacial dilatational rheology. Aiming to re-create the oleosome interface, we isolated the oleosome membrane and after dispersing it in water, allowed it to diffuse on an oil/water interface. First, we measured the interfacial tension *γ* as a function of the bulk concentration of the oleosome membrane PL/oleosin mixture (Fig. 4(a)). The analysis showed that by increasing the concentration in the bulk phase, the interfacial tension was decreased, indicating that a higher number of molecules was adsorbed at the interface (higher surface density). Even higher concentrations (0.05 wt%) led to a rapid decrease of the interfacial tension (*γ* < 3 mN m^−1^) and spontaneous expelling of the droplet (data not shown here).

To study the molecular lateral interactions concerning the molecular density at the interface, we further applied dilatational interfacial rheology, where we determined the elastic (
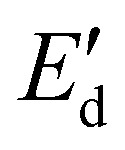
) and viscous (
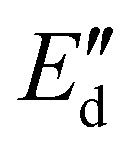
) moduli of the interface as a function of the amplitude of deformation as presented in Fig. 4(b). The 
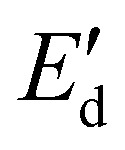
 was higher than the 
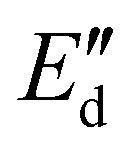
 and ranged between 18 and 20 mN m^−1^ in all concentrations tested. These 
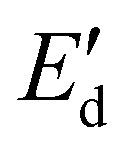
 values are relatively low compared to, for example, interfaces stabilized by proteins that interact at the interface (*i.e.*, whey protein isolate 
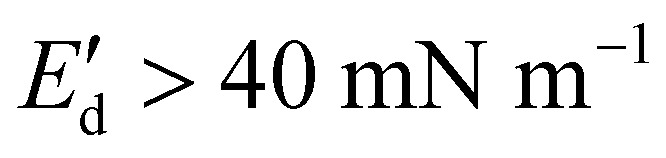
),^37^ suggesting the absence of strong network formation at the interface. At low bulk concentrations (0.001 wt%), we noted a slight decrease of 
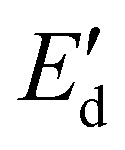
 for increasing amplitude of deformation.

At the highest concentration (0.01 wt%), we could not apply amplitudes above 15%, as upon compression, the increase in molecular density and crowding of molecules led to the expelling of the droplet from the syringe. However, by lowering the concentration to 0.005 wt% or 0.001 wt%, we could compress the interface to up to 30% and 50%, respectively, before expelling the droplet.

Aiming to dive deeper into the molecular lateral interactions at the interface, we used Lissajous curves, where we plotted the surface pressure (*γ* − *γ*_0_) against the area deformation (Δ*A*/*A*_0_) at the highest amplitude that we could achieve at each concentration (Fig. 4(c)). At the highest concentration (0.01 wt%) and 15% amplitude, the curve had a narrow elliptical shape, implying a primarily elastic response of the interface.^38^ Upon maximum compression (lower left part) and subsequent expansion, the curve has a pointy tip, meaning a similar response in surface pressure upon compression and subsequent expansion, indicating weak lateral interactions.^37^ Lateral interactions may originate: (1) from PL polar head groups or the hydrophilic domain of the LD-associated proteins through electrostatic and/or van der Waals forces and (2) from PL tail groups or the hydrophobic parts of the proteins. However, the main type of PL in oleosomes is phosphatidylcholine,^39^ which is zwitterionic and limits the interactions between polar heads, while PL tail–tail interactions are counteracted by PL tail–TAG interactions.^40^ In addition, oleosins, which are the main proteins present on the oleosome interface, have a very small hydrophilic part and an extended hydrophobic part.^41^ The hydrophobic domain would strongly interact with the lipids on the droplet, hampering further protein–PL interactions. A previous study on the interfacial properties of oleosins has also demonstrated that oleosins do not extensively interact at the interface, forming a weak network that can be disrupted upon oscillations.^42^

By lowering the bulk concentration (0.005 wt%) and reaching 30% amplitude, the shape of the plot remained elliptical but slightly wider (more viscous), while we observed non-linearities; upon expansion (upper right part), the curve started to slightly bend horizontally, showing interfacial strain softening. This softening behavior eventuates from disruption (weakening) of the molecular network at the interface upon higher expansion of the droplet surface area. As more interface was created, fewer molecules per area were present (increase in surface gaps), leading to an increase in the surface pressure. The results are also in line with the changes in PL interactions, clustering, and increase in area per PL upon lipid absorption and volume expansion of oleosomes, as observed in MD simulations.

At the same concentration (0.005 wt%) upon compression (lower left part), the curve was slightly bent vertically. This result suggests strain stiffening behavior on compression, probably due to the formation of densely clustered regions^43^ (highly packed interface). PL monolayers have been found to form a tangential lattice at close packing and become oriented more normally to the interface at lower concentrations^40^ (*e.g.*, extension). The strain hardening due to increased molecular density per interfacial area might also explain the reorganization of the adsorbed molecules on the interface into a liquid-crystalline shell. Both strain softening on expansion and strain hardening on compression were even more apparent in the Lissajous curve of the lowest concentration tested (0.001 wt%), as the lower molecular density at the interface allowed even higher deformations (50% amplitude). Overall, this behavior upon expansion (softening) and compression (hardening) indicates the formation of a 2d soft glass phase at the interface^38^ and shows weak lateral interactions which can be disrupted, facilitating the dilation of the oleosome interface.

### An *in silico* investigation of the distribution of phospholipids on the oleosome interface upon expansion of the available surface

Molecular dynamics (MD) simulations are a powerful tool that can provide further insights into the intermolecular interactions within the oleosome interface.^44,45^ Performing MD simulations enable us to suggest and elaborate on the possible physical mechanisms governing the incorporation of added oil into oleosomes and the subsequent redistribution of the membrane molecules on the available surface. To achieve that, we used the coarse-grained Martini force field^23^ simulation of a “model” with only PL on the surface. Proteins can also play an important role in the lateral interactions on the oleosome interface, but initially, we aimed to show the interactions between the PLs. To simulate the oil phase, we used triolein as a model triacylglycerol^46^ and DPPC as a simple type of PL. It is important to note that the melting temperature of DPPC described by the Martini force field is lower than 283 K,^31^ which is substantially lower than the experimentally measured melting temperature (314 K). Thus, in all simulations performed here, DPPC remained in the liquid phase, and no aggregation or clustering of the DPPC molecules on the surface was observed.^23^ Similar results are expected if other PLs with unsaturated fatty acid chains were used. The Martini force field is a natural choice here because it is proven to be an indispensable tool for simulating lipid bilayers, and a fully atomistic representation of the molecules would limit the MD simulations to timescales at which oil incorporation would not occur.^47,48^

**Fig. 5:**
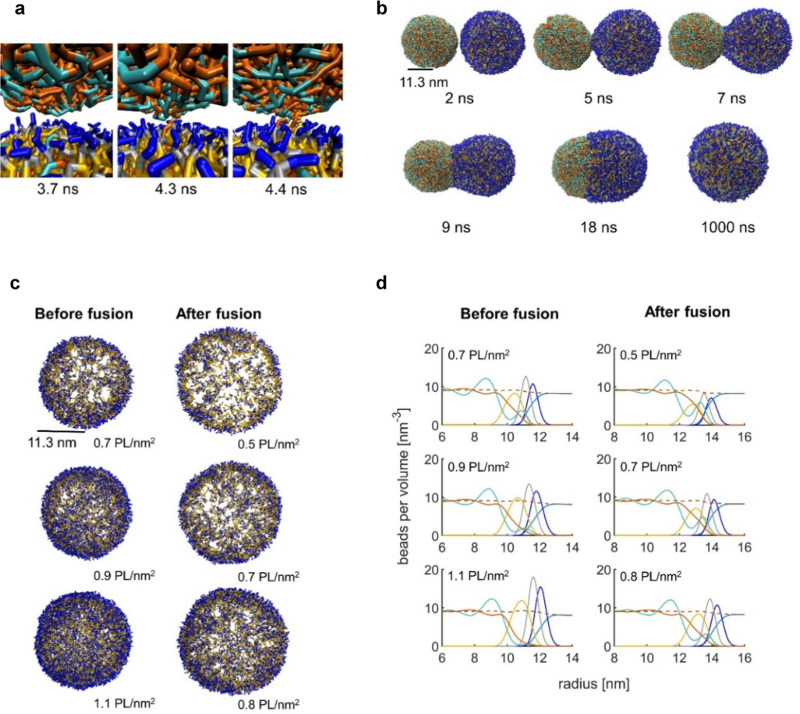
(a) Snapshots of MD simulations showing the initial contact of oleosomes having 0.9 PL nm^−2^. The slightly hydrophilic heads of the TAG molecules are shown in cyan color, while the hydrophobic tails are shown in orange color. The color code used for the DPPC molecules is as follows: blue color for the head groups, grey color for the glycerol sections, and yellow color for the tails. Water molecules are omitted for clarity. (b) Simulation snapshots showing the time evolution of the fusion process. (c) Oleosomes of different PL densities showing the PL distribution before and after fusion, and the increase in oleosome size after fusion (only DPPC molecules are shown). (d) Radial component density graphs of oleosomes (left) and oleosomes fused with the TAG assemblies (right). Colors: PL glycerol group (grey), PL head groups (blue), PL lipid groups (yellow), TAG glycerol group (cyan), and TAG hydrophobic tails (orange). The bead positions used to construct the radial component density are sampled from 1000 simulation frames sampled every 10 ps. The simulation details can be found in the Methods section.

Initially, we created configurations of assembled triolein molecules and covered them with PLs with a varied PL density from 0.7 to 1.1 PL per nm^2^, following rough surface coverage calculations based on the reported concentrations of PLs in oleosomes^17–19^ (Table S1, ESI†). We then placed a “free” triolein droplet next to the model oleosomes and investigated the mechanism of the incorporation of triolein and its coverage with the available PL (Fig. 5). This is a different mechanism compared to the homogenization we applied during our experiments, where we disrupt and reform the membrane, but nevertheless it is useful to get insights into the interactions of the interfacial PL with the free TAGs.

As shown in Fig. 5(a) despite the dense packing of the PL on the interface (0.9 PL nm^−2^), it is still not fully covered because the free TAGs could still have access to the TAGs in the core and through hydrophobic forces, they were able to diffuse towards the core. The presence of gaps on the PL-covered interface has been suggested earlier for a density of 1 PL per 7 nm^2^,^49^ but complete coverage is not achieved even with 6 times denser surface. Initially, to come into proximity, the neighboring droplets should overcome the energy barrier associated with the hydration repulsion,^50^*i.e.* the force acting between the two solvated droplets keeping them apart. As time evolves, the TAGs of the assembly create a contact site with the surface of the oleosomes through the interstitial water film.^51^ This contact is initially triggered by the attractive hydrophobic forces between the free TAGs and the area with PL hydrophobic tails embedded in the core TAGs. After the first contact, a stalk is formed, entailing interfacial deformations (Fig. 5(b)), which specifically requires the protrusion of the hydrophobic PL tail of oleosomes towards the free TAGs.^50^ The stalk in turn expands to create a fusion pore and subsequently a channel, showing the mobility of the PLs on the surface and their ability to rearrange and lay over when in contact with the external TAGs. An animation of the MD simulation of this fusion progress is provided in the ESI.†

The lateral interactions might be affected by the density and packing of the PL on the oleosome interface, therefore we investigated whether the PL will react in a similar way when surface density will be higher and up to 1.1 PL nm^−2^ (Fig. 5(c)). From the droplets shown in Fig. 5(c) (left column), it is clear that at 0.7 PL nm^−2^, there is more void space at the interface, which reduces by increasing the number of PL to 0.9 and 1.1 PL nm^−2^. Statistical analysis of our simulations showed that the likelihood of the contact of the free TAGs with the core of oleosomes decreased as the number of PL per interfacial area increased. In particular, for oleosomes with 0.7 PL nm^−2^, the probability of TAG incorporation was found to be 0.70 ± 0.14 (*i.e.*, 7 out of 10 simulations resulted in infused systems), while for oleosomes with 0.9 and 1.1 PL nm^−2^, the probability of TAG incorporation was 0.50 ± 0.16 and 0.30 ± 0.14, respectively. The standard deviations were calculated based on the assumption that the fusion process can be approximated by a binomial distribution.^52^ The results of this experimental design show that when the PLs are more packed on the membrane, they leave fewer gaps for the TAGs to be in contact with the bulk, however, PL molecules do not form strong clusters and the PL fatty acids are still solubilized in the TAGs of the core, having very weak lateral interactions. Upon stimuli, which in this case is the external hydrophobic material, the PL can still lay over and change conformation, forming a tunnel.

After incorporating the free TAGs in the model oleosomes, the surface of the droplets increased (Table S1, ESI†), leading to a less dense interface compared to the initial system (Fig. 5(c) right column), where the PLs were redistributed, covering the entire surface in a somehow homogeneous manner.

To better understand and quantify the arrangement of the PLs on the oleosome interface and their interaction with the TAGs, we computed the respective radial component densities (RCDs). Fig. 5(d) shows the RCDs, illustrating the distribution of the PL and lipid molecules over the radius of oleosomes before and after the incorporation of additional TAGs. In the cases with low interfacial density (0.7 PL nm^−2^), the TAGs glycerol group (cyan line) is placed towards the surface and overlaps with the PL glycerol (grey line) and head groups (blue line) (Fig. 5(d) left column). This orientation is imposed by the voids at the droplet interface, causing the more hydrophilic part of the TAG molecule (glycerol) to move forward to limit the unfavourable interaction of the TAG hydrophobic tails with water. With increasing interfacial density (*i.e.* decreasing the surface voids), the molecular orientation of the TAG changes in such a way that their hydrophobic tails (orange line) move towards the surface to interact with the PL glycerol (grey line) and lipid groups (yellow line). Despite the changes in the orientation of the core TAGs, the fatty acids of the PLs are not in direct contact and no strong interactions take place. As a result, when the surface area increases, the PLs start redistributing, reaching a new equilibrium.

### The phospholipids on the oleosome membrane can reorient upon an external trigger and release the contained oil

**Fig. 6:**
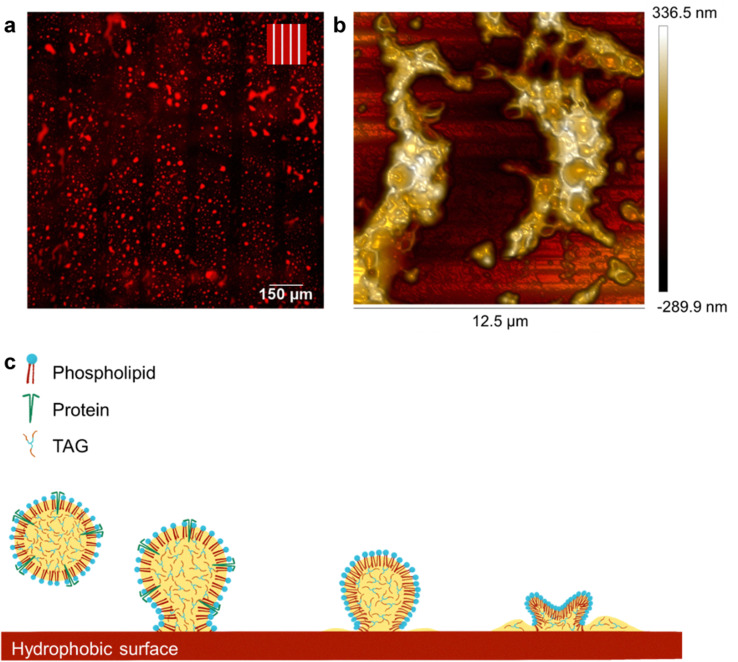
(a) Fluorescent LM images of the purified oleosomes stained with Nile Red on the surface of alternating hydrophobic (150 μm broad) and hydrophilic (50 μm broad) lanes. Scale bar 150 μm. In the schematic representation of the surfaces, red and light blue represent the hydrophobic and hydrophilic part, respectively, (b) 3D AFM images of oleosomes on a modified hydrophobic surface, (c) graphical visualization of the hypothetical mechanism of lipid release from oleosomes on the hydrophobic surface, showing the protrusion of PL tails towards the hydrophobic surface to establish contact, and the subsequent shrinking of oleosomes (schematic representations not to scale).

The molecular simulation data on the rearrangement of the membrane PL, when the free TAGs were in contact with the core TAGs, inspired us to use it as a way to reorient the surface PL and open a channel, so maybe the inner oil can be released. Therefore, we functionalized hydrophobic glass surfaces and deposited oleosome dispersions on them. Despite the glass surface being negatively charged, the same as oleosomes, we observed visually an adhesion of oleosomes on the surface. The adhesive forces were probably originating in hydrophobic forces, but to clarify that, we functionalized alternating hydrophobic and hydrophilic lanes, and after the deposition of an oleosome dispersion, we gently rinsed with water. As shown in the fluorescent light microscopy image in Fig. 6(a), the red fluorescent oleosomes were not interacting at all with the lanes that were hydrophilic (empty black stripes) but were concentrated on the hydrophobic lanes. Despite the extensive rinsing with water, they could not be removed, showing a strong adhesion.

The light microscope observations indicated that the oleosomes were clustered and possible free oil was present, therefore, we used atomic force microscopy (AFM) to investigate in detail the type of clusters on the surface (Fig. 6(b)). The analysis revealed the presence of clustered oleosomes with a retained spherical periphery (greater height) and a sunken hub (lower height), surrounded by bulk material, probably free lipids (greater height). Probably, despite the coverage of oleosomes with PLs and proteins that expose their hydrophilic domains, it is possible that there are some hydrophobic voids on the surface, which interacted with the hydrophobic surface. Similar to what was observed with the molecular simulations, it is possible that the relatively mobile PL changed conformation due to hydrophobic interactions, opening bigger voids that allowed the release of the inner TAGs. As oleosomes did not rupture and only deflated upon this contact, we suggest that the release of lipids was realized through the creation of a channel due to the PL reorganization towards the hydrophobic interface, as shown in Fig. 6(c). As the oleosome lipid core was released through this channel, oleosomes shrink, and to reduce the membrane tension arising from the very high molecular density (crowding) in the interface, they finally buckle and deflate.

## Conclusions

In this work, we investigated the lateral interactions of the molecules in the oleosome (lipid droplet) membrane. Using experimental soft matter science combined with coarse-grained molecular dynamics simulations, we showed that the oleosome membrane is dilatable, with only weak interactions between the membrane molecules. When dispersed oleosomes in water are homogenised in the presence of free lipids, the oleosome membrane molecules redistribute, adjusting their interfacial molecular density on the new available interface reaching a new equilibrium. This behaviour was irrelevant to the surface density and the initial packing of the PL and was also observed for extremely dense interfaces with 1 PL per nm^2^. According to the molecular simulations, TAGs interact with the PL fatty acids and “solubilize” them, preventing PL clustering. When free TAGs were attached to the membrane, the PLs changed orientation and lay over to form a channel and redistribute and cover the free TAGs as well. The weak interactions between the oleosome membrane molecules and the “mobility” of the PL could be exploited to form channels on the oleosome interface through external hydrophobic forces and release their oil core. We anticipate that our findings could contribute to a better understanding of the behaviour of oleosomes in dispersions and their potential use as stimuli-responsive carriers of hydrophobic molecules. It would be interesting to further investigate the interactions of the inner oil with the membrane PL and proteins and how the lateral interactions are effected by the chemistry of the oil.

## Conflicts of interest

There are no conflicts to declare.

## Supplementary Material

SM-019-D3SM00449J-s001
