# Supplementary material for: The dilatable membrane of oleosomes (lipid droplets) allows their in vitro resizing and triggered release of lipids
Source: Soft Matter. 2023 Aug 5;19(33):6355–67. doi: 10.1039/d3sm00449j (PMC10445523; doi:10.1039/d3sm00449j)
Supplement: SM-019-D3SM00449J-s001 [file SM-019-D3SM00449J-s001.pdf]

This journal is © The Royal Society of Chemistry 2023

Appendix A: Supplementary material

Composition analysis

To determine the moisture content of the recovered purified oleosomess, samples (~1.00 g) were dried at 60°C until reaching a stable weight (24 hrs). The samples cooled down to room temperature in a glass desiccator (Duran, Wertheim/Main, Germany) for 30 min. The moisture content (wt.%) was calculated based on the weight loss after drying.

The lipid content (LC) of the oleosomes was calculated on a dry-matter weight basis using Soxhlet extraction. The lipids were extracted for 7 hrs with petroleum ether (40-60°C) as a solvent. The lipid content after extraction was calculated using:

$$LC\ (wt\%) = 100 * \left(\frac{M_o}{M}\right)$$

Equation 1S

where M_o [g] is the mass of the extracted TAGs.

The protein content (PC) of the recovered purified oleosomes on dry-matter weight basis was determined using the dumas method (FlashEA 1112 Series, Thermo Scientific, Waltham, Massachusetts, US); d-methionine (≥98%, Sigma Aldrich, Darmstadt, Germany) was used as a standard and as a control. Cellulose (Sigma Aldrich, Darmstadt, Germany) served as blank. A nitrogen–protein conversion factor of 5.7 (calculated based on amino acid sequence) was used and the protein content was calculated using:

$$PC\ (wt\%) = 100 * \left(\frac{NC * 5.7}{M}\right)$$

Equation 2S

where PC is the protein content, NC is the nitrogen content, and M is the mass of the dry sample.

Electrophoresis (SDS-PAGE)

The protein profile of the proteins in purified oleosomes was determined with SDS-PAGE. The samples were analyzed under non-reducing and reducing conditions. Reducing agent (NuPAGE ® Sample Reducing Agent) was added to break disulphide bonds in napin and cruciferin chains, enabling the detection of their presence. The samples were prepared as follows:

- 100 µL sample with a protein concentration of 3.3 mg/mL on average.
- 250 µL NuPAGE ® LDS sample buffer
- 100 µL NuPAGE ® Sample Reducing Agent or deionized water
- 550 µL deionized water

The samples were vortexed and then centrifuged for one minute at 2000 rpm to eliminate undissolved material. Subsequently, samples were heated in a heating block (Eppendorf Thermomixer C, Eppendorf Nederland B.V., Nijmegen, the Netherlands) for 10 minutes at 70°C to denature the proteins. Samples were centrifuged at the same settings again.

18 µL of sample were loaded in a NuPAGE Novex® (by Thermo Fischer SCIENTIFIC, Walham, USA) gel (4-12% Bis-Tris, 1.0mm, 12 wells), submersed in a NuPAGE® MES SDS running buffer. 10 µL of a PageRuler™ Plus prestained protein ladder (10-250 kDa) was loaded. The gels ran for a minimum of 35 minutes at a constant 200 V in a Mini Gel Tank (Invitrogen by Fischer Scientific, Waltham, USA).

Subsequently, the gels were rinsed three times with demi-water and stained with Coomassie Brilliant Blue R-250 staining solution for 50 minutes while gently shaking at room temperature. The gels were rinsed three times with demi-water and destained with washing buffer (10 wt% ethanol and 7.5 wt% acetic acid in deionized water) for a minimum of two hours at room

temperature. Afterwards, the gels were stored at room temperature in demi-water filled plastic boxes. The lids were covered with aluminum foil to prevent light degradation of the bands.

Results on characterization of purified rapeseed oleosomes

Protein profile

To evaluate the purity of the obtained oleosomes the protein profile was analyzed using electrophoresis (SDS-PAGE). The electophoregram (**Figure 1**) shows the protein profile of the oleosomes under non-reducing conditions. The oleosins (15-17 kDa), caleosins (20-27 kDa) and steroleosins (40-55 kDa) appeared to constitute the majority of proteins present. Above 115 kDa some undefined bands were present, which may have been a slight carry-over of enzymes[15]. Almost no bands related to storage rapeseed proteins (napins and cruciferins) were present, indicating a relatively pure system.

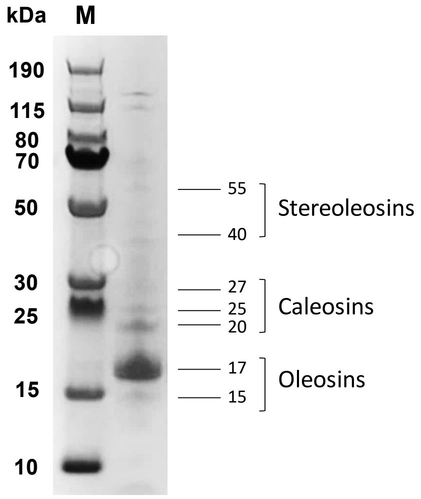

Figure 1S. Protein profile of purified oleosomes under non-reducing conditions. M: protein molecular weight marker.

Molecular dynamic simulations

Table 1S Measured radii (*r*) PL per Area (nm^2) of oleosome-like droplets and Area (nm^2) per PL of oleosome-like droplets of 1200-2000 DPPC molecules per oleosome before and after fusion.

**Droplet**	**r [nm]**	**PL/nm^2**	**nm^2/PL**
Triolein assembly	10.3	-	-
Oleosome-1200	11.3	0.7	1.34
Oleosome-1600	11.6	0.9	1.06
Oleosome-2000	12.0	1.1	0.90
Oleosome-1200 fused	13.6	0.52	1.93
Oleosome-1600 fused	13.8	0.67	1.50
Oleosome-2000 fused	14.2	0.79	1.27
